# Combined Application of Ultra-High-Pressure Homogenization and Non-*Saccharomyces* Yeasts to Reduce Sulfites and Improve Wine Quality

**DOI:** 10.3390/foods15132271

**Published:** 2026-06-25

**Authors:** Maria Soler, Carmen Gonzalez, Antonio Morata, Iris Loira

**Affiliations:** Department of Chemistry and Food Technology, Escuela Técnica Superior de Ingeniería Agronómica, Alimentaria y de Biosistemas, Universidad Politécnica de Madrid, 28040 Madrid, Spain; maria.soler@alumnos.upm.es (M.S.); carmen.gchamorro@upm.es (C.G.); antonio.morata@upm.es (A.M.)

**Keywords:** oenology, ultra-high-pressure homogenization (UHPH) non-*Saccharomyces*, *Lachancea thermotolerans*, *Metschnikowia pulcherrima*

## Abstract

In recent years, the wine industry has searched for alternatives to reduce the use of sulfites, with ultra-high-pressure homogenization (UHPH) emerging as a promising technology. The objective of this study was to evaluate the combined effect of UHPH and non-*Saccharomyces* yeasts (*Lachancea thermotolerans* and *Metschnikowia pulcherrima*) on wine quality. To this end, fermentations were carried out using control Verdejo must, must treated with UHPH, and must treated with 50 mg/L SO_2_, using pure cultures and co-inoculations. Enological parameters, volatile compounds, colour, redox potential, and sensory profile were analyzed. The results showed that the co-inoculation of *L. thermotolerans* and *M. pulcherrima* reduced the final ethanol content by 0.5% (*v*/*v*) and increased lactic acid production, resulting in a decrease in pH of 0.3 units; however, *L. thermotolerans* in monoculture failed to implant properly in UHPH-treated must. In addition, wines from UHPH-treated musts exhibited 20% lower redox potential, suggesting that treatment with UHPH was effective in inactivating oxidative enzymes, as did those fermented with *M. pulcherrima*. Regarding volatile compounds, UHPH wines showed a 30% reduction in higher alcohols and carbonyl compounds, resulting in a characteristic aromatic profile that was positively evaluated in sensory analysis. In conclusion, the combination of UHPH and non-*Saccharomyces* yeasts represents an effective strategy for improving wine quality and reducing the use of sulfites.

## 1. Introduction

Recently, there has been growing interest in reducing the use of additives such as sulfites in the wine industry. Their use can compromise wine quality by neutralizing aromas or causing the formation of undesirable aromas. In addition, high concentrations of sulfites carry certain health risks, such as nausea, headaches, stomach irritation, and respiratory problems in sensitive individuals [1,2]. In this context, new alternative technologies have emerged, such as the application of ultra-high-pressure homogenization (UHPH) to must.

UHPH is a technology capable of fragmenting particles suspended in a liquid when it is subjected to high pressures (200–600 MPa) through the passage of a valve [3]. This fragmentation is caused by shear forces, turbulence, and cavitation [4]. These forces also affect the microorganisms and spores present in the must, producing sterilization [5]. As it is not a thermal method, UHPH protects the sensory profile without affecting pigments or aromas [6]. Likewise, an inactivating effect on oxidative enzymes (polyphenol oxidase, PPOs) has been observed, which has a protective effect against oxidation and browning in white wines [7,8].

Due to its sterilizing capacity, UHPH is a very interesting technology to use in combination with non-*Saccharomyces* yeasts, which can present problems of implantation and low tolerance to sulfites. Due to their metabolic differences, non-*Saccharomyces* yeasts are a great strategy for adding diversity to wines or enhancing the aromatic profile of neutral varieties [9]. In this trial, two non-*Saccharomyces* yeasts were used: *Lachancea thermotolerans* (Lt) and *Metschnikowia pulcherrima* (Mp).

Lt is an acidifying yeast capable of producing up to 15 g/L of lactic acid under oenological conditions [10]. Lactic acid is a product of sugar metabolism, making it also interesting for reducing alcohol content [11,12,13]. An increase in the production of 2-phenylethyl acetate, with a rose petal aroma, has also been observed, which also improves the sensory profile [14].

Mp is of interest for its ability to produce pulcherriminic acid, which has an antimicrobial effect [15,16], and for its oxygen consumption, which reduces oxidative stress [17]. It also promotes the release of varietal aroma precursors, such as thiols and monoterpenes [18,19,20,21].

The objective of this study was to evaluate the combined effect of UHPH technology and inoculation with non-*Saccharomyces* yeasts as an alternative strategy in winemaking. This approach aims to reduce reliance on sulfites by exploiting the ability of UHPH to inactivate oxidative enzymes, while improving the chemical and sensory profiles of the wine thanks to the metabolic diversity of non-*Saccharomyces* yeast strains.

## 2. Materials and Methods

### 2.1. Strains Used in Fermentation

Three yeast strains, previously cultured in YPD liquid medium, were used for the fermentation of Verdejo grape must. Two successive passages were performed in YPD medium, inoculating 2% of the volume of the previous culture, for 48 h at 28 °C under constant agitation (120 rpm). Following this process, the cultures were verified by plating on YPD agar plates to confirm that they had reached a population of between 10^7^ and 10^8^ CFU/mL.

The must was inoculated by adding a volume of liquid culture (in YPD medium) equivalent to 2% of the total fermentation volume. The strains *Lachancea thermotolerans* BLIZZ™ (Lallemand Inc., Montreal, QC, Canada) and *Metschnikowia pulcherrima* M29 were evaluated individually and in mixed co-inoculation. In the co-inoculation, they were inoculated simultaneously in a 1:1 ratio, adding 2% (*v*/*v*) of each culture. The *Saccharomyces cerevisiae* 7 VA strain (EnotecUPM, Madrid, Spain) was used as the fermentation control. The initial population was confirmed by microbiological counting, ensuring it was around 10^6^ CFU/mL in the must.

### 2.2. Verdejo Must

Fermentation was carried out with two types of Verdejo grape must: a control and one treated with UHPH at 3000 bar for 0.2 s, without exceeding 102 °C. Both musts come from a single batch of Verdejo grapes from the Comenge winery (Curiel del Duero, Valladolid, Spain), which was divided into two parts for the UHPH treatment. The grapes were harvested mechanically, and the musts were racked separately, pressed with a pneumatic press, and clarified at 4 °C. Before inoculation, Fourier transform infrared (FTIR) spectroscopy (OenoFOSS, FOSS Iberia, Barcelona, Spain) was used to determine the concentration of reducing sugars, total acidity expressed as tartaric acid, and nitrogen assimilable by yeast (Table 1).

### 2.3. Must Fermentation

**Figure 1:**
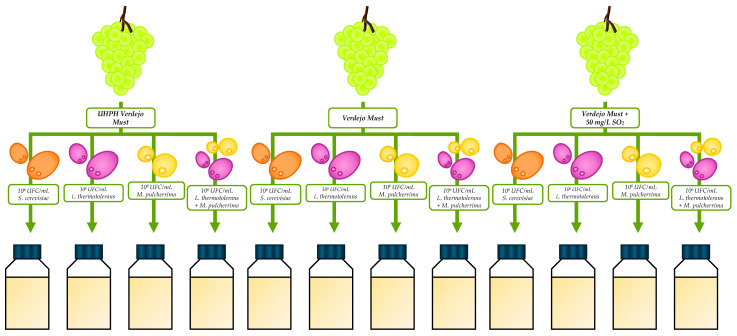
Fermentation conditions for the trials (all in triplicate).

The effect of UHPH technology was evaluated by comparing parallel fermentations in 250 mL volumes using untreated Verdejo musts, musts processed by UHPH, and musts treated with 50 mg/L of SO_2_ (Figure 1). Fermentations were carried out at 20°C with *Saccharomyces cerevisiae* and sequential fermentations with *Lachancea thermotolerans*, *Metschnikowia pulcherrima*, or a co-inoculation of *L. thermotolerans* with *M. pulcherrima,* following the experimental design described below. All tests were performed in triplicate. On the 7th day of fermentation, *S. cerevisiae* 7VA was inoculated using the same inoculation protocol to complete the fermentation of sugars in those musts that had initially been inoculated with non-*Saccharomyces* yeasts.

Periodically, general oenological parameters were analyzed using FTIR, pH was measured, and lactic acid concentration was determined by enzymatic analysis. At the end of fermentation, redox potential and colorimetric parameters were evaluated, and a sensory analysis was performed.

### 2.4. Analysis of General Oenological Parameters

Daily, the pH evolution was determined using the FTIR optical system to track the kinetic evolution of fermentation and indirectly indicate yeast implantation. This system estimates pH through a mathematical prediction model based on mid-infrared absorption spectra. However, due to interference from CO_2_, ethanol and turbidity during active fermentation, these values may vary slightly in absolute precision compared to the baseline measurements obtained with a standard pH meter before fermentation. At the end of fermentation, FTIR analysis was performed to determine the ethanol content (% *v*/*v*), residual sugar content (g/L), volatile acidity (g/L acetic acid) and malic acid content (g/L). Final pH was determined using a pH 80 bench pH meter (XS Instruments, Carpi, Italy).

### 2.5. Enzyme Analysis

To evaluate the acidifying capacity of *L. thermotolerans*, lactic acid concentrations were determined using the A25 clinical chemistry analyser (Biosystems, Barcelona, Spain) using BioSystem’s L-lactic acid analysis kit.

### 2.6. Colour Analysis

Measurements of absorbance at 420, 520 and 620 nm, colour intensity, chroma, hue and CIELab coordinates were performed using the Smart Analysis spectrophotometer (DNA Phone, Parma, Italy) using a 1 cm path-length plastic cuvette. Before analysis, all samples were filtered through a 0.45 µm membrane filter to prevent light scattering caused by turbidity.

The colour of the samples was represented in CIELab coordinates, characterizing the colours based on the coordinates L*, a* and b*. The L* coordinate represents the brightness and can take values between 0 (black) and 100 (white). The a* coordinate can take negative (green) or positive (red) values. The b* coordinate indicates the position between blue (negative) and yellow (positive).

From the L*a*b* coordinates, chroma (C*) and hue (H*) parameters were obtained. The hue values represent red (0°), yellow (90°), green (180°), and blue (270°), and the chroma is proportional to the colour saturation, with grey represented by 0.

All the parameters were computed automatically by the instrument’s software.

### 2.7. Determination of Redox Potential

To determine the redox potential at the end of fermentation, the optical potentiometer with the HI73120 electrode (Hanna Instruments, Eibar, Guipuzkoa, Spain) was used.

### 2.8. Analysis of Fermentative Volatile Compounds

Analysis of fermentative volatile compounds was conducted on an Agilent Technologies 6850 gas chromatograph (Las Rozas, Madrid, Spain), integrated with a flame ionization detector (GC-FID) and a DB-624 column (60 m × 250 μm × 1.40 μm) according to the method described by [22].

### 2.9. Sensory Analysis

A descriptive sensory analysis evaluated visual, olfactory and taste parameters. The parameters were scored using a scale from 1 (lowest intensity) to 5 (highest intensity). The parameters analysed were colour intensity, tonality, turbidity, aromatic intensity, aromatic quality, herbs, floral, fruity, reduction and oxidation aromas, body, silkiness, bitterness, acidity and overall perception. The taste panel of trained experts who analysed the wines consisted of eight members of the Food Technology laboratory (ETSIAAB), including both genders and ages between 20 and 50 years old. All evaluators provided voluntary informed consent before participation. The sensory analysis took place in a tasting room with artificial lighting, with 30 mL of wine served per glass. The samples were tasted once in a blind tasting, and the order of the samples was randomized. The experts cleansed their palates between samples using breadsticks and water. This descriptive sensory evaluation method is an adaptation of the International Organization for Standardization (ISO) guidelines to the conditions of our research [23,24]. The study was conducted in accordance with the Declaration of Helsinki and approved by the Institutional Review Board (UPM Ethics Committee) of Universidad Politécnica de Madrid (AID-LAPPMLB-AMB-HUMANOS-20221026) on 14 November 2022.

### 2.10. Statistical Analysis

Statistical analyses were performed using RStudio 2024.9.0 (RStudio Team, Boston, MA, USA). Data are expressed as means and standard deviations. The results were subjected to one-way Analysis of Variance (ANOVA), and means were compared using Tukey’s test. Statistical significance was established at *p* < 0.05. Additionally, principal component analysis (PCA) and graphical representations were generated using the same software.

## 3. Results and Discussion

### 3.1. General Oenological Parameters

Table 2 summarizes the general oenological parameters analyzed at the end of fermentation. The ethanol content ranged between 12.3% and 15.7% (*v*/*v*), with significantly higher values (*p* < 0.05) in wines fermented from control must, consistent with the higher initial sugar concentration in this must. The potential of *L. thermotolerans* to produce wines with lower alcohol content has been previously reported and is a result of sugars being diverted to lactic acid production [11,12,13]. Although no reduction in ethanol content was observed in wines fermented solely with *L. thermotolerans*, wines co-inoculated with *L. thermotolerans* and *M. pulcherrima* exhibited a lower ethanol concentration.

Regarding residual sugar, no differences were observed among wines as a function of must type. However, wines fermented with non-*Saccharomyces* yeasts tended to show higher residual sugar levels compared to those fermented with *Saccharomyces cerevisiae*. Notably, one of the control wines co-inoculated with *L. thermotolerans* and *M. pulcherrima* did not complete fermentation, resulting in a mean final residual sugar concentration of 5 g/L. This may occur because the *L. thermotolerans* and *M. pulcherrima* strains used have a fermentation capacity of 9 g/L of ethanol and 4 g/L of ethanol, respectively, and the *S. cerevisiae* inoculated on the seventh day of fermentation may not have established itself under optimal conditions to finish consuming the sugars.

Volatile acidity ranged from 0.05 to 0.23 g/L acetic acid, very low values that are not associated with sensory defects and are consistent with those obtained in fermentations under similar conditions [25]. Concerning must type, differences were observed in wines fermented with *S. cerevisiae*, which showed higher volatile acidity in UHPH-treated musts and in wines fermented with *L. thermotolerans*, which exhibited lower volatile acidity when *L. thermotolerans* failed to implant, as can be seen from the absence of lactic acid production. In the control must, where *L. thermotolerans* was successfully implanted, wines showed increased volatile acidity. However, co-inoculation with *L. thermotolerans* and *M. pulcherrima* effectively reduced volatile acidity. This effect is due to the stronger respiratory metabolism of *M. pulcherrima* during early fermentation, which consumes oxygen and limits acetic acid synthesis, reducing volatile acidity [26].

Regarding pH, wines produced from UHPH-treated must and SO_2_-treated must exhibited lower pH values than those fermented from control must. pH and lactic acid concentration were also useful indicators of the implantation success of *L. thermotolerans*. In control must, *L. thermotolerans* successfully implanted both in monoculture and in co-inoculation, producing 2.9 g/L of lactic acid and decreasing pH by 0.3 units compared to wines fermented with *S. cerevisiae*. The absence of lactic acid production in UHPH-treated must and must treated with 50 mg/L SO_2_ suggests that *L. thermotolerans* did not ferment properly, potentially due to nutritional deficiency and sulfite concentrations exceeding the strain’s tolerance threshold (10–20 mg/L of free SO_2_) [27], respectively. Nevertheless, successful implantation occurred in co-inoculation with *M. pulcherrima*, with the highest lactic acid production observed in UHPH-treated must (4 g/L) and a corresponding pH reduction of 0.4 units.

With respect to malic acid, wines produced from UHPH-treated must exhibited lower malic acid concentrations. Additionally, a further decrease was observed in wines fermented with *L. thermotolerans*. This difference may be attributed to the capacity of certain *L. thermotolerans* strains to metabolize malic acid during fermentation [12].

### 3.2. pH Evolution

**Figure 2:**
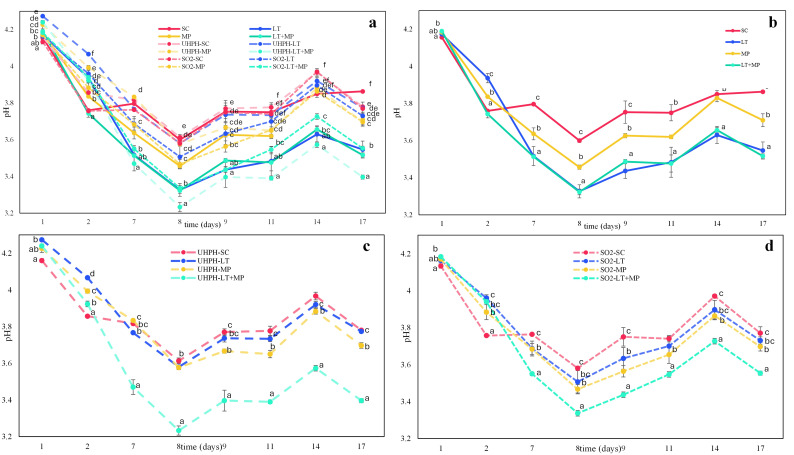
Time course of pH during fermentation. Wines fermented with *S. cerevisiae* are shown in red, those fermented with *L. thermotolerans* in blue, those fermented with *M. pulcherrima* in yellow and the co-inoculation of *L. thermotolerans* and *M. pulcherrima* in cyan. The solid line represents the wines made with the control must, the dashed line represents the wines made with the UHPH must and the dotted line represents the wines made with the control treated with 50 mg/L of SO_2_. The graph shows the evolution of all conditions (**a**) and broken down by category: the evolution of wines fermented from control must (**b**), wines fermented from UHPH must (**c**), and wines fermented from must treated with 50 mg/L of SO_2_ (**d**). Values between which there is no significant difference (*p* > 0.05) according to Tukey’s test are indicated with the same letter.

The pH of the samples was measured daily during fermentation (Figure 2). At the beginning of fermentation, pH values ranged from 4.14 to 4.28. On the second day, differences in pH began to appear between fermentations carried out with different yeast strains. By the seventh day, significant differences (*p* < 0.05) were observed in fermentations where *L. thermotolerans* successfully implanted, including control musts inoculated with *L. thermotolerans*, co-inoculations with *L. thermotolerans* and *M. pulcherrima* in control must, and co-inoculations in UHPH-treated must and must treated with 50 mg/L SO_2_. These fermentations exhibited pH values approximately 0.3 units lower than their counterparts fermented with *S. cerevisiae*. From day 9 onward, pH values stabilized. This rapid decrease in pH during the first week coincides with the exponential growth phase of *L. thermotolerans*, a period during which lactic acid is actively synthesized from sugars via lactate dehydrogenase [28]. From day 9 onward, pH values stabilized. This stabilization reflects the stop in lactic acid production, likely due to the accumulation of ethanol, which limits the viability of *L. thermotolerans* [25], as well as the sequential inoculation of *S. cerevisiae* on day 7, which shifts the metabolic process towards primary alcoholic fermentation without further acidification.

The results showed that wines produced from UHPH-treated must co-inoculated with *L*. *thermotolerans* and *M*. *pulcherrima* presented the lowest pH (3.397 ± 0.012), coinciding with the highest lactic acid production (4.0 ± 1.2 g/L). The second-lowest pH values were observed in wines fermented with *L. thermotolerans* in control must, as well as in co-inoculations of *L. thermotolerans* and *M. pulcherrima* in control must and in must treated with 50 mg/L SO_2_, with pH values around 3.5 and lactic acid concentrations ranging from 2.3 to 2.5 g/L.

The higher lactic acid production observed in coinoculated fermentations is consistent with previous reports describing a strong synergistic effect between *L. thermotolerans* and *M. pulcherrima* [11].

### 3.3. Colourimetric Analysis

Spectrophotometric analysis revealed significant differences in several colour parameters among the samples at a significance level of 0.05 (Table 3).

In terms of colour intensity (CI), the sum of absorbances at 420, 520, and 620 nm, wines fermented with *S. cerevisiae* exhibited significantly higher intensity than their counterparts, with statistically significant differences particularly evident in wines produced from must treated with 50 mg/L SO_2_. This greater CI is likely due to the antioxidant and protective effects of SO_2_ against the oxidation of polyphenols.

Regarding tonality, an indicator of the degree of browning in wines, no significant differences were observed among the samples.

For chroma, another parameter associated with colour intensity, higher values were detected in wines produced from UHPH-treated must. Additionally, greater chroma values were observed in wines fermented with *S. cerevisiae* and in those co-inoculated with *L. thermotolerans* and *M. pulcherrima* from UHPH-treated and SO_2_-treated musts.

In relation to hue, no significant differences were found between the different samples.

Brightness, expressed by the L* coordinate, showed significantly higher values in wines fermented with non-*Saccharomyces* yeasts, highlighting the antioxidant effect of *M. pulcherrima*, which prevents oxidation and therefore preserves wine brightness through the production of pulcherriminic acid. Wines produced from must treated with 50 mg/L SO_2_ also exhibited the highest brightness values, although these differences were not statistically significant.

Concerning the CIELab coordinates a* and b*, no significant differences were found for a*, with all samples displaying values close to zero, indicating the absence of pronounced green or red tonalities. In contrast, b* values followed a trend similar to chroma, with higher values observed in wines fermented from UHPH-treated must, reflecting a more intense yellow coloration. Increased yellow intensity was also detected in wines fermented with *S. cerevisiae* and in co-inoculations with *L. thermotolerans* and *M. pulcherrima* from UHPH-treated and SO_2_-treated musts.

### 3.4. Redox Potential Analysis

Redox potential was measured using an optical sensor. The results showed that wines produced from UHPH-treated must exhibited the lowest redox potential. This could suggest lower oxidative activity, potentially linked to the mitigation of oxidative enzymes such as polyphenol oxidase (PPO) during the pressure treatment [25]; however, further direct enzymatic analyses would be required to confirm this mechanism. The wines with the next-lowest potential were those fermented from must treated with 50 mg/L of SO_2_, which aligns with the well-known reducing and antioxidant properties of sulfites.

**Figure 3:**
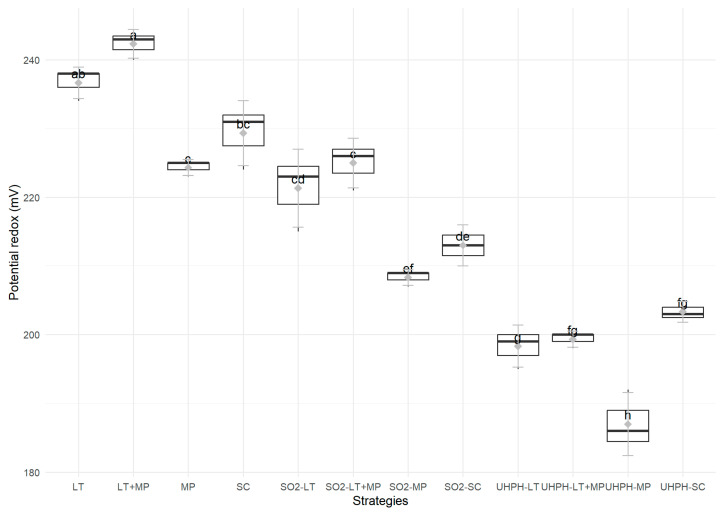
Boxplot of redox potential at the end of fermentation. SC, LT, MP, and LT + MP correspond to wines made from control must fermented with *S. cerevisiae*, *L. thermotolerans*, *M. pulcherrima*, and the co-inoculation of *L. thermotolerans* + *M. pulcherrima*, respectively. UHPH-SC, UHPH-LT, UHPH-MP, and UHPH-LT + MP correspond to wines obtained from must treated with UHPH and fermented with the indicated yeasts. SO_2_-SC, SO_2_-LT, SO_2_-MP, and SO_2_-LT + MP correspond to wines from control must treated with 50 mg/L SO_2_ and fermented with the indicated yeasts. Values between which there are no significant differences (*p* > 0.05) according to Tukey’s test are indicated with the same letter.

Regarding fermentative yeasts, a decrease in redox potential was observed in wines fermented with *M. pulcherrima* (Figure 3), highlighting its antioxidant capacity [26]. This effect is thought to be related to the intense respiratory metabolism of *M. pulcherrima* during the early stages of fermentation, which leads to rapid consumption of dissolved oxygen and creates a more reducing environment. In contrast, wines fermented with *L. thermotolerans* showed a higher redox potential.

### 3.5. Volatile Analysis

Table 4 presents the volatile compounds produced during fermentation, quantified by GC-FID. Total volatile concentrations ranged from approximately 1100 to 1700 mg/L. Fermentations carried out using UHPH-treated must exhibited lower total volatile production, which may be explained by its reduced nitrogen content [29,30]. Among the wines fermented from UHPH must, those fermented with *M. pulcherrima*, as well as those produced by co-inoculation of *L. thermotolerans* and *M. pulcherrima*, showed total volatile concentrations of 950 mg/L and 980 mg/L, respectively, compared to 1150 mg/L in wines fermented with *S. cerevisiae*. Conversely, among wines produced from must treated with sulfites, wines fermented with *M. pulcherrima* showed a higher volatile production than those fermented with *S. cerevisiae*.

Although UHPH-treated must resulted in an overall lower volatile content, these differences were mainly attributable to the reduced formation of carbonyl compounds and higher alcohols.

Regarding carbonyl compounds, an approximate 15% reduction in 2,3-butanediol production was observed in wines fermented from UHPH-treated must. This compound is a by-product of alcoholic fermentation with a slightly sweet–sour taste and no significant impact on aroma [31]. No significant differences were found among yeast strains within the same must. Higher acetaldehyde production was detected in wines fermented from UHPH must, tripling the levels observed in control must and doubling those found in SO_2_-treated must. This effect may be attributed to the low nitrogen availability in the must [32,33]. In terms of yeast species, non-*Saccharomyces* strains were able to reduce acetaldehyde production. No differences were observed in the concentrations of other carbonyl compounds.

With respect to higher alcohols, wines fermented from UHPH-treated must exhibited significantly lower concentrations, remaining below 400 mg/L, a threshold above which negative sensory effects may occur [34]. This reduction could be explained by the fact that, without constituting a stress factor for the yeast, the lower nitrogen content in the UHPH must may have provided fewer precursors for the Ehrlich pathway, which is involved in the synthesis of higher alcohols; alternatively, the higher nitrogen content in the other musts may have allowed for greater biomass growth, potentially leading to more vigorous fermentation kinetics [35,36]. However, non-*Saccharomyces* yeasts showed higher production of 1-propanol, isobutanol, 3-methyl-1-butanol, and 2-methyl-1-butanol. Concerning 2-phenylethanol, a compound associated with rose-like floral aromas, concentrations ranged between 10 and 14 mg/L, exceeding the sensory threshold of 10 mg/L [37]. Higher levels were found in wines fermented with *L. thermotolerans*, as previously reported in the literature [38].

Acetate esters are formed through the esterification of acyl-CoA with alcohols and, with the exception of ethyl acetate, are generally associated with fruity aromas and positive sensory contributions [39]. All wines contained ethyl acetate concentrations below 150 mg/L, the level above which solvent-like aromas may develop [40]. Despite the lower nitrogen content of UHPH must, ester production was not negatively affected, potentially due to the inactivation of oxidative enzymes. Regarding 2-phenylethyl acetate, which contributes rose-like floral notes, all fermentation strategies produced concentrations above the perception threshold (0.25 mg/L) [41]. Wines fermented with *L. thermotolerans* showed the highest levels, except for those produced from UHPH-treated must, likely due to poor yeast implantation, as evidenced by the absence of lactic acid production.

**Figure 4:**
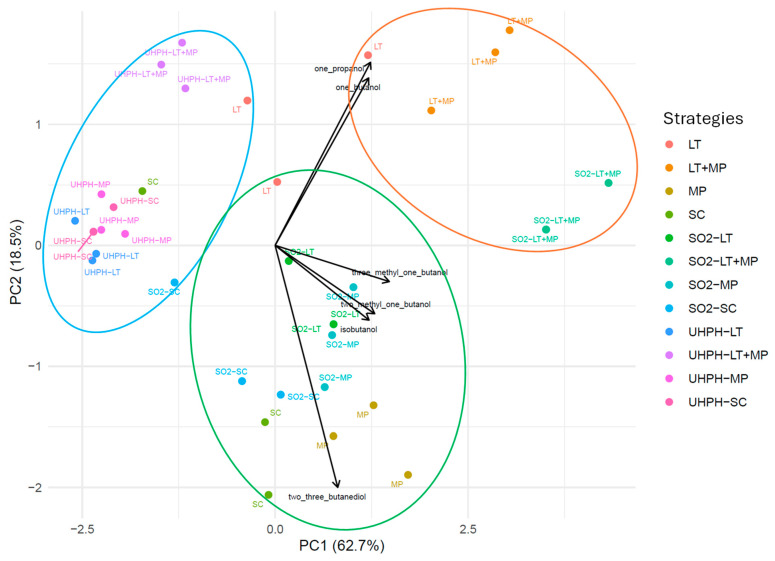
Principal component analysis performed on the concentrations of volatile compounds measured with GC-FID. SC, LT, MP, and LT + MP correspond to wines made from control must fermented with *S. cerevisiae*, *L. thermotolerans, M. pulcherrima*, and the co-inoculation of *L. thermotolerans + M. pulcherrima*, respectively. UHPH-SC, UHPH-LT, UHPH-MP, and UHPH-LT + MP correspond to wines obtained from must treated with UHPH and fermented with the indicated yeasts. SO_2_-SC, SO_2_-LT, SO_2_-MP, and SO_2_-LT + MP correspond to wines from control must treated with 50 mg/L SO_2_ and fermented with the indicated yeasts.

A principal component analysis (PCA) was conducted using volatile compounds with factor loadings greater than 0.7. The first two principal components explained 81.2% of the total variance (Figure 4). Three main clusters were identified: a blue cluster comprising wines produced from UHPH-treated must, which separated clearly along PC1 due to their lower higher-alcohol production; a red cluster grouping wines fermented through *L. thermotolerans* and *M. pulcherrima* co-inoculation; and a green cluster including wines fermented with pure cultures of *S. cerevisiae*, *L. thermotolerans* and *M. pulcherrima* from control and SO_2_-treated must.

### 3.6. Variable Correlation

**Figure 5:**
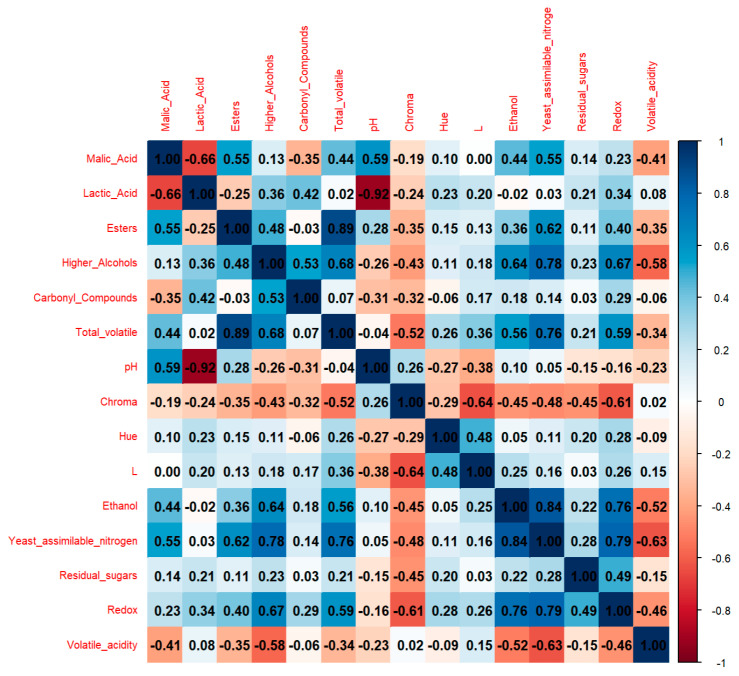
Correlation matrix indicating Pearson’s coefficients calculated from the measured parameters.

Based on the collected data, a correlation matrix was constructed using Pearson’s correlation coefficients to assess relationships among the evaluated variables (Figure 5). This coefficient ranges from −1 to 1, with values further from zero indicating stronger correlations. Positive values indicate direct relationships between variables, whereas negative values reflect inverse correlations.

The results indicated that pH differences were primarily driven by variations in lactic acid concentration. A positive correlation was also observed between nitrogen content, higher-alcohol composition, total volatile composition, and redox potential. This relationship can be explained by the fact that higher nitrogen availability provides more precursors for the Ehrlich pathway, thereby enhancing higher-alcohol synthesis and increasing total volatile production.

The correlation observed with redox potential may be attributed to the inactivation of oxidative enzymes as a consequence of the UHPH treatment.

### 3.7. Sensory Analysis

**Figure 6:**
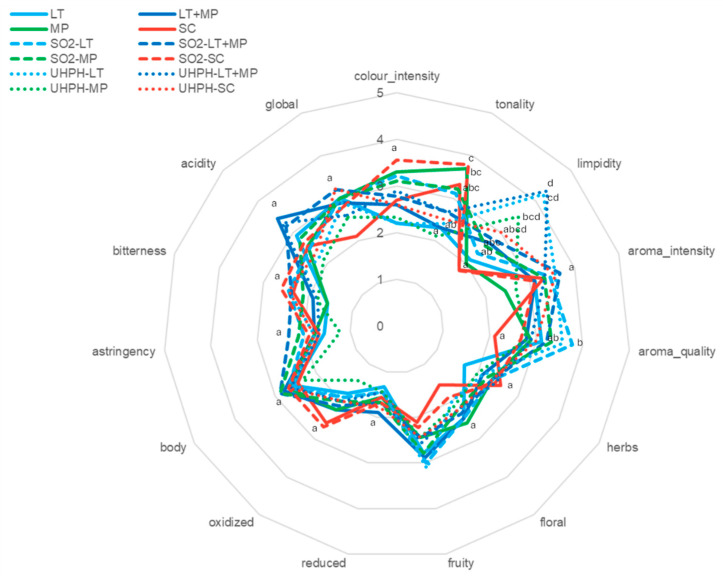
Spider graph showing the results of the sensory analysis carried out by eight judges. Wines fermented with *S. cerevisiae* are shown in red, those fermented with *L. thermotolerans* in blue, those fermented with *M. pulcherrima* in green and the co-inoculation of *L. thermotolerans* and *M. pulcherrima* in cyan. The solid line represents the wines made with the control must, the dashed line represents the wines made with the UHPH must and the dotted line represents the wines made with the control treated with 50 mg/L of SO_2_. Values between which there is no significant difference (*p* > 0.05) according to Tukey’s test are indicated with the same letter.

Sensory analysis revealed significant differences in the perception of hue, clarity, and aromatic quality, with no significant differences among judges’ evaluations (Figure 6). Overall, the wines exhibited an acidic and astringent profile, with sweeter and fruitier aromas perceived in wines fermented with non-*Saccharomyces* yeasts.

Regarding visual attributes, judges assigned low scores for tonality, indicating a greenish tone and limited oxidation. Wines produced from UHPH-treated must showed lower tonality values, with significant differences observed in the fermentation strategy involving *M. pulcherrima*. This effect may be attributed to a combination of the antioxidant action of the UHPH treatment and the protective effect against oxidation associated with *M. pulcherrima*. In addition, judges rated wines fermented from UHPH-treated must higher in clarity, suggesting that this process contributes to protein stabilization and facilitates clarification [26,42]. This does not agree with the colorimetry data, although it should be noted that the visible spectrophotometry was performed on the day fermentation ended (under reducing conditions), so the colour may have evolved further by the time of the sensory analysis.

In the evaluation of olfactory attributes, wines fermented with *L. thermotolerans* received the highest scores, whereas wines fermented with *S. cerevisiae* received the lowest scores and were described by judges as more oxidized. This may be related to the fact that, although there are no significant differences, wines fermented with *S. cerevisiae* tend to score higher on the “oxidized” parameter, which also corresponds to higher concentrations of acetaldehyde.

Finally, regarding taste perception, although no statistically significant differences were detected, wines produced by co-inoculation of *L. thermotolerans* and *M. pulcherrima* were perceived as more acidic.

## 4. Conclusions

The results of the trial indicated that co-inoculation of Lt and Mp was an effective strategy for modulating wine composition. In particular, this combination reduced the final ethanol content and promoted lactic acid production, with a consequent decrease in pH. However, when Lt was used in monoculture in UHPH-treated must, adequate implantation was not achieved during fermentation. Unfortunately, the physicochemical differences between the musts prevented us from delving deeper into the matter.

Likewise, a reduction in redox potential was observed in wines made from UHPH-treated must, which could be related to the inactivation of oxidative enzymes during treatment. A similar decrease in redox potential was also detected in wines fermented with Mp.

In terms of volatiles, wines fermented from UHPH must had a characteristic volatile profile, with lower levels of carbonyl compounds and higher alcohols. This profile was rated positively in sensory analysis, indicating that the application of UHPH, in combination with non-*Saccharomyces* yeasts, can contribute to improving the sensory quality of wine.

The use of UHPH technology in combination with non-*Saccharomyces* yeasts represents a promising strategy in modern winemaking, helping to modulate the wine’s sensory profile and control the oxidative environment in order to preserve the wine’s freshness.

## Figures and Tables

**Table 1:** Physicochemical parameters of the musts used for fermentation.

	Control Must	UHPH Must
Reducing sugars (g/L)	264.3	220.3
pH *	3.87	3.82
Total acidity expressed as tartaric acid (g/L)	3.44	2.52
Total SO_2_ (mg/L)	<5	50
Yeast-assimilable nitrogen (mg/L)	275.7	225.5

* All parameters were measured by FT-IR, except for pH, which was determined using a pH meter.

**Table 2:** General oenological parameters at the end of fermentation. Values between which there is no significant difference (*p* > 0.05) according to Tukey’s test are indicated with the same letter. Ethanol content, pH, glucose plus fructose, and volatile acidity were determined using infrared spectroscopy. The concentrations of malic acid and lactic acid were measured by enzymatic analysis. Values are expressed as mean ± standard deviation (n = 3).

Strategy	Ethanol (%vol)	pH	Glucose Plus Fructose (g/L)	Volatile Acidity (g/L Acetic Acid)	Malic Acid (g/L)	Lactic Acid (g/L)
*S. cerevisiae*	14.9 ± 0.4 ^bcd^	3.863 ± 0.006 ^f^	0.4 ± 0.4 ^a^	0.08 ± 0.01 ^ab^	3.00 ± 0.17 ^def^	0.107 ± 0.012 ^a^
*L*. *thermotolerans*	15.7 ± 0.5 ^d^	3.55 ± 0.05 ^b^	4.3 ± 1.4 ^ab^	0.26 ± 0.05 ^d^	1.8 ± 0.4 ^b^	2.9 ± 0.6 ^bc^
*M. pulcherrima*	15.5 ± 0.3 ^cd^	3.71 ± 0.03 ^cd^	2.5 ± 0.3 ^a^	0.163 ± 0.015 ^bcd^	3.03 ± 0.11 ^ef^	0.113 ± 0.015 ^a^
*L. thermotolerans + M. pulcherrima*	14.5 ± 0.3 ^c^	3.52 ± 0.01 ^b^	5.0 ± 3.0 ^b^	0.223 ± 0.023 ^d^	2.4 ± 0.6 ^abcde^	2.9 ± 0.13 ^bc^
UHPH-*S. cerevisiae*	13.0 ± 0.3 ^a^	3.78 ± 0.00 ^e^	0.0 ± 0.0 ^a^	0.197 ± 0.025 ^cd^	2.3 ± 0.0 ^bcd^	0.11 ± 0.01 ^a^
UHPH-*L*. *thermotolerans*	13.2 ± 0.4 ^a^	3.773 ± 0.006 ^de^	1.0 ± 1.6 ^a^	0.103 ± 0.006 ^abc^	2.1 ± 0.12 ^bc^	0.133 ± 0.005 ^a^
UHPH-*M. pulcherrima*	12.3 ± 0.5 ^a^	3.697 ± 0.015 ^c^	3.0 ± 2.0 ^ab^	0.170 ± 0.017 ^bcd^	2.63 ± 0.15 ^cde^	0.10 ± 0.01 ^a^
UHPH-*L. thermotolerans + M. pulcherrima*	12.4 ± 0.3 ^a^	3.397 ± 0.012 ^a^	1.0 ± 1.7 ^a^	0.23 ± 0.06 ^d^	0.93 ± 0.15 ^a^	4.0 ± 1.2 ^c^
SO_2_-*S. cerevisiae*	15.1 ± 0.2 ^bcd^	3.77 ± 0.03 ^de^	1.0 ± 1.4 ^ab^	0.053 ± 0.015 ^a^	2.87 ± 0.06 ^def^	0.09 ± 0.00 ^a^
SO_2_-*L*. *thermotolerans*	15.3 ± 0.2 ^bcd^	3.73 ± 0.02 ^cde^	3.7 ± 2.1 ^ab^	0.10 ± 0.04 ^abc^	3.47 ± 0.12 ^d^	0.15 ± 0.04 ^a^
SO_2_-*M. pulcherrima*	15.5 ± 0.4 ^cd^	3.70 ± 0.02 ^c^	2.4 ± 0.4 ^a^	0.153 ± 0.005 ^abcd^	3.03 ± 0.15 ^fe^	0.17 ± 0.04 ^a^
SO_2_-*L. thermotolerans + M. pulcherrima*	14.4 ± 0.3 ^b^	3.55 ± 0.01 ^b^	0.9 ± 0.9 ^a^	0.23 ± 0.08 ^d^	2.33 ± 0.06 ^abcd^	2.26± 0.12 ^b^

**Table 3:** Colourimetric parameters of the wine samples analysed. Values between which there is no significant difference (*p* > 0.05) according to Tukey’s test are indicated with the same letter. Values are expressed as mean ± standard deviation (n = 3).

Strategy	Colour Intensity (Absorbance Units)	Tonality (Adimensional)	Chroma	Hue (°)	L	a	b
*S. cerevisiae*	0.80 ± 0.13 ^ab^	1.57 ± 0.21 ^a^	12 ± 3 ^ab^	87.3 ± 1.2 ^a^	82 ± 4 ^ab^	0.56 ± 0.09 ^a^	13 ± 3 ^abc^
*L*. *thermotolerans*	0.42 ± 0.14 ^a^	1.67 ± 0.23 ^a^	10 ± 3 ^ab^	87 ± 4 ^a^	91 ± 3 ^b^	0.5 ± 0.6 ^a^	10 ± 3 ^ab^
*M. pulcherrima*	0.27 ± 0.09 ^a^	2.4 ± 0.3 ^a^	10.0 ± 0.8 ^ab^	93.0 ± 1.8 ^a^	94.9 ± 2.3 ^b^	−0.52 ± 0.26 ^a^	10.0 ± 0.8 ^abc^
*L. thermotolerans + M. pulcherrima*	0.48 ± 0.12 ^a^	1.60 ± 0.03 ^a^	7.9 ± 0.6 ^a^	92 ± 5 ^a^	89 ± 3 ^ab^	−0.3 ± 0.7 ^a^	7.8 ± 0.7 ^a^
UHPH-*S. cerevisiae*	0.9 ± 0.3 ^ab^	1.75 ± 0.32 ^a^	14.5 ± 1.3 ^b^	88 ± 4 ^a^	82 ± 7 ^ab^	0.5 ± 1.0 ^a^	14.5 ± 1.3 ^bc^
UHPH-*L. thermotolerans*	0.52 ± 0.20 ^a^	1.9 ± 0.5 ^a^	13.3 ± 1.4 ^ab^	87 ± 4 ^a^	89 ± 5 ^b^	0.6 ± 0.9 ^a^	13.2 ± 1.4 ^abc^
UHPH-*M. pulcherrima*	0.7 ± 0.6 ^ab^	1.62 ± 0.21 ^a^	13 ± 5 ^ab^	85 ± 3 ^a^	85 ± 13 ^ab^	1.4 ± 1.2 ^a^	13 ± 5 ^abc^
UHPH-*L. thermotolerans + M. pulcherrima*	0.5 ± 0.3 ^a^	2.3 ± 0.8 ^a^	14.3 ± 1.6 ^b^	90 ± 6 ^a^	89 ± 8 ^b^	0.0 ± 1.4 ^a^	14.2 ± 1.5 ^bc^
SO_2-_*S. cerevisiae*	1.3 ± 0.2 ^b^	1.52 ± 0.10 ^a^	15.8 ± 0.9 ^b^	87 ± 3 ^a^	71 ± 5 ^a^	0.8 ± 0.9 ^a^	15.7 ± 0.9 ^c^
SO_2-_*L*. *thermotolerans*	0.38 ± 0.08 ^a^	1.8 ± 0.4 ^a^	11.7 ± 1.5 ^ab^	87 ± 3 ^a^	91.6 ± 2.0 ^b^	0.5 ± 0.6 ^a^	11.7 ± 1.5 ^abc^
SO_2-_*M. pulcherrima*	0.30 ± 0.03 ^a^	1.659 ± 0.022 ^a^	10.2 ± 0.3 ^ab^	86.1 ± 1.3 ^a^	93.1 ± 0.8 ^b^	0.7 ± 0.2 ^a^	10.2 ± 0.3 ^abc^
SO_2-_*L. thermotolerans + M. pulcherrima*	0.35 ± 0.05 ^a^	2.4 ± 0.7 ^a^	12.9 ± 0.9 ^ab^	91 ± 4 ^a^	93.3 ± 1.6 ^b^	−0.25 ± 0.9 ^a^	12.9 ± 0.9 ^abc^

**Table 4:** Quantification of volatile fermentation compounds using GC-FID. Values between which there is no significant difference (*p* > 0.05) according to Tukey’s test are indicated with the same letter. Values are expressed as mean ± standard deviation (*n* = 3).

	Compounds (mg/L)	SC	LT	MP	LT + MP	UHPH–SC	UHPH–LT	UHPH–MP	UHPH–LT + MP	SO_2_–SC	SO_2_-LT	SO_2_–MP	SO_2_–LT + MP
Carbonyl compounds	acetaldehyde	120 ± 30 ^ab^	60 ± 30 ^a^	43 ± 12 ^a^	87 ± 11 ^a^	300 ± 80 ^c^	260 ± 30 ^c^	100 ± 50 ^bc^	205 ± 9 ^bc^	210 ± 30 ^bc^	90 ± 30 ^a^	50 ± 20 ^a^	140 ± 40 ^ab^
	diacetyl	1.540 ± 0.170 ^a^	1.920 ± 0.240 ^a^	1.860 ± 0.240 ^a^	1.860 ± 0.240 ^a^	1.830 ± 0.040 ^a^	1.575 ± 0.023 ^a^	1.630 ± 0.050 ^a^	1.900 ± 0.300 ^a^	1.640 ± 0.120 ^a^	1.630 ± 0.070 ^a^	2.000 ± 0.500 ^a^	1.890 ± 0.021 ^a^
	acetoin	10.0 ± 4.0 ^a^	7.1 ± 0.6 ^a^	9.0 ± 1.0 ^a^	9.2 ± 1.4 ^a^	9.6 ± 1.6 ^a^	9.0 ± 1.1 ^a^	9.1 ± 1.6 ^a^	10.9 ± 0.8 ^a^	11.0 ± 5.0 ^a^	12.7 ± 0.3 ^a^	10.6 ± 2.2 ^a^	11.0 ± 4.0 ^a^
	2,3-butanediol	700 ± 300 ^ab^	430 ± 40 ^ab^	710 ± 40 ^b^	590 ± 40 ^ab^	540 ± 40 ^ab^	490 ± 40 ^ab^	510 ± 50 ^ab^	390 ± 110 ^a^	600 ± 120 ^ab^	646 ± 14 ^ab^	600 ± 60 ^ab^	710 ± 30 ^b^
Higher alcohols	1-propanol	30.000 ± 4.000 ^a^	61.000 ± 8.000 ^d^	45.600 ± 2.200 ^bc^	77.500 ± 2.700 ^e^	35.747 ± 0.015 ^ab^	30.700 ± 1.400 ^a^	39.000 ± 4.000 ^abc^	46.500 ± 1.000 ^c^	29.600 ± 2.300 ^a^	44.550 ± 0.170 ^bc^	40.900 ± 2.700 ^bc^	74.000 ± 5.000 ^e^
	isobutanol	29.0 ± 4.0 ^a^	70.0 ± 6.0 ^b^	182.0 ± 24.0 ^e^	100.0 ± 5.0 ^c^	27.1 ± 2.4 ^a^	29.0 ± 4.0 ^a^	49.0 ± 5.0 ^ab^	42.0 ± 4.0 ^a^	30.2 ± 1.7 ^a^	70.0 ± 6.0 ^b^	126.0 ± 12.0 ^d^	163.0 ± 3.0 ^e^
	1-butanol	4.40 ± 0.30 ^a^	4.80 ± 0.70 ^a^	4.05 ± 0.05 ^a^	6.80 ± 0.90 ^b^	4.30 ± 0.01 ^a^	3.92 ± 0.06 ^a^	4.02 ± 0.18 ^a^	4.92 ± 0.24 ^a^	4.30 ± 0.40 ^a^	4.90 ± 0.40 ^a^	4.66 ± 0.22 ^a^	6.60 ± 0.50 ^b^
	3-methyl-1-butanol	183.0 ± 21.0 ^cd^	222.0 ± 15.0 ^de^	237.0 ± 15.0 ^e^	255.0 ± 15.0 ^e^	85.0 ± 14.0 ^a^	91.7 ± 0.4 ^ab^	77.0 ± 7.0 ^a^	127.0 ± 9.0 ^b^	180.0 ± 18.0 ^c^	221.0 ± 14.0 ^de^	229.0 ± 10.0 ^e^	297.0 ± 11.0 ^f^
	2-methyl-1-butanol	92.00 ± 4.00 ^bc^	83.00 ± 17.00 ^bc^	92.00 ± 13.00 ^bc^	103.00 ± 4.00 ^cd^	42.00 ± 8.00 ^a^	52.02 ± 0.22 ^a^	47.00 ± 6.00 ^a^	66.80 ± 1.80 ^ab^	108.00 ± 12.00 ^cd^	96.00 ± 22.00 ^cd^	99.00 ± 10.00 ^cd^	122.30 ± 1.60 ^d^
	hexanol	3.70 ± 0.30 ^a^	4.00 ± 0.30 ^a^	3.62 ± 0.05 ^a^	3.80 ± 0.08 ^a^	3.90 ± 0.30 ^a^	3.72 ± 0.24 ^a^	3.63 ± 0.25 ^a^	3.77 ± 0.10 ^a^	3.58 ± 0.18 ^a^	3.78 ± 0.17 ^a^	3.72 ± 0.20 ^a^	3.86 ± 0.25 ^a^
	2-phenylethanol	11.60 ± 1.70 ^ab^	14.20 ± 0.60 ^bc^	12.10 ± 0.80 ^abc^	12.66 ± 0.16 ^abc^	11.20 ± 0.80 ^ab^	10.60 ± 0.90 ^a^	11.60 ± 0.60 ^ab^	14.60 ± 1.20 ^c^	12.40 ± 1.10 ^abc^	12.60 ± 1.10 ^abc^	11.30 ± 0.60 ^ab^	13.73 ± 0.05 ^bc^
	**Total**	350 ± 30 ^b^	480 ± 70 ^de^	570 ± 50 ^e^	551 ± 12 ^b^	204 ± 23 ^a^	216 ± 6 ^a^	228 ± 18 ^a^	303 ± 10 ^ab^	360 ± 30 ^bc^	450 ± 40 ^cd^	509 ± 10 ^de^	672 ± 17 ^f^
Esters	isobutyl acetate	1.500 ± 1.700 ^a^	0.400 ± 0.700 ^a^	1.257 ± 0.010 ^a^	1.700 ± 0.500 ^a^	2.800 ± 0.900 ^a^	1.200 ± 1.000 ^a^	1.200 ± 1.200 ^a^	1.420 ± 0.070 ^a^	1.600 ± 0.600 ^a^	0.500 ± 0.900 ^a^	0.900 ± 0.700 ^a^	1.517 ± 0.021 ^a^
	ethyl butyrate	2.60 ± 1.20 ^a^	2.00 ± 0.60 ^a^	1.70 ± 0.30 ^a^	1.48 ± 0.18 ^a^	1.50 ± 0.21 ^a^	1.90 ± 0.30 ^a^	4.00 ± 5.00 ^a^	3.00 ± 3.00 ^a^	1.65 ± 0.15 ^a^	1.80 ± 0.30 ^a^	1.59 ± 0.14 ^a^	1.67 ± 0.22 ^a^
	ethyl lactate	38.0 ± 10.0 ^b^	25.0 ± 14.0 ^ab^	13.0 ± 3.0 ^ab^	21.0 ± 5.0 ^ab^	11.0 ± 4.0 ^a^	26.0 ± 10.0 ^ab^	32.0 ± 20.0 ^ab^	10.0 ± 3.0 ^a^	17.0 ± 13.0 ^ab^	12.0 ± 4.0 ^ab^	8.4 ± 1.5 ^a^	19.0 ± 6.0 ^ab^
	isoamyl acetate	2.40 ± 0.60 ^a^	2.22 ± 0.03 ^a^	2.27 ± 0.22 ^a^	2.57 ± 0.17 ^a^	2.20 ± 0.70 ^a^	2.40 ± 0.50 ^a^	2.50 ± 0.40 ^a^	2.60 ± 0.30 ^a^	2.51 ± 0.23 ^a^	2.26 ± 0.23 ^a^	2.16 ± 0.18 ^a^	2.25 ± 0.27 ^a^
	2-phenylethyl acetate	5.80 ± 0.40 ^b^	6.50 ± 0.30 ^ab^	7.20 ± 0.80 ^ab^	8.20 ± 2.20 ^ab^	6.00 ± 0.80 ^a^	5.63 ± 0.01 ^a^	5.80 ± 0.30 ^a^	8.90 ± 1.10 ^b^	7.40 ± 1.70 ^ab^	6.10 ± 0.70 ^ab^	7.60 ± 0.80 ^ab^	7.00 ± 0.80 ^ab^
	**Total**	63 ± 10 ^a^	90 ± 24 ^a^	54 ± 11 ^a^	72 ± 7 ^a^	54 ± 10 ^a^	84 ± 13 ^a^	70 ± 30 ^a^	75 ± 16 ^a^	41 ± 11 ^a^	51 ± 7 ^a^	70 ± 30 ^a^	70 ± 14 ^a^
	methanol	30.0 ± 15.0 ^ab^	41.0 ± 8.0 ^abc^	42.0 ± 5.0 ^abc^	47.8 ± 1.7 ^bc^	38.7 ± 2.0 ^abc^	42.0 ± 4.0 ^abc^	31.0 ± 5.0 ^a^	36.9 ± 0.9 ^a^	42.0 ± 4.0 ^abc^	49.0 ± 3.0 ^c^	44.0 ± 3.0 ^abc^	48.9 ± 1.4 ^c^
	**Total**	1200 ± 400 ^ab^	1110 ± 90 ^ab^	1440 ± 70 ^bc^	1370 ± 40 ^abc^	1150 ± 80 ^ab^	1110 ± 30 ^ab^	950 ± 90 ^a^	980 ± 50 ^a^	1300 ± 170 ^abc^	1300 ± 70 ^abc^	1300 ± 30 ^abc^	1660 ± 80 ^c^

## Data Availability

The original contributions presented in this study are included in the article. Further inquiries can be directed to the corresponding author.

## Abbreviations

The following abbreviations are used in this manuscript:

UHPH	ultra-high-pressure homogenization
Sc	*Saccharomyces cerevisiae*
Lt	*Lachancea thermotolerans*
Mp	*Metschnikowia pulcherrima*
